# Reaction of Nitrate Radicals and DMPO in Non‐Aqueous Media: A Novel Detection Tool to Highlight the Nitrate Radical Role in Photocatalytic Processes

**DOI:** 10.1002/anie.202505313

**Published:** 2025-04-02

**Authors:** Alessia Zollo, Alessandro Gottuso, Leonardo Palmisano, Elio Giamello, Francesco Parrino, Stefano Livraghi

**Affiliations:** ^1^ Department of Chemistry and NIS Centre University of Torino Via Pietro Giuria 7 Torino 10125 Italy; ^2^ Department of Industrial Engineering University of Trento Via Sommarive 9 Trento 38123 Italy; ^3^ Department of Engineering University of Palermo Viale delle Scienze 9, Ed. 6 Palermo 9012 Italy

**Keywords:** DMPO, EPR spectroscopy, Nitrate radicals, TiO_2_ photocatalysis

## Abstract

The reaction of DMPO as the spin trapping agent with nitrate radicals photocatalytically generated results in the formation of DMPOX showing a highly specific EPR spectrum. The method can be used as a semi‐quantitative and convenient way to detect nitrate radicals in non‐aqueous solutions/suspensions, which was never achieved up to now through EPR spectroscopy. Results support in a more direct way the proposed generation and reactivity of nitrate radicals in photocatalytic media and open a new chapter in the field of heterogeneous photocatalysis.

## Graphical abstract

The spin trapping agent DMPO reacts with nitrate radicals in non‐aqueous environment producing its oxidized adduct DMPOX. The peculiar EPR spectral features of DMPOX can be considered fingerprint of the presence of nitrate radicals. Results support in a more direct way the proposed generation and reactivity of nitrate radicals in heterogeneous photocatalysis.

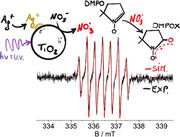

The formation of nitrate radicals (NO_3_^•^) in solution garnered increasing attention due to their relevance in atmospheric and environmental chemistry,^[^ ^1^ ^]^ but more recently in nuclear solvent extraction,^[^ ^2^ ^]^ and in photochemical/photocatalytic applications.^[^ ^3^ ^]^ The direct spectroscopic observation of nitrate radicals is particularly difficult,^[^ ^4^ ^]^ and to the best of our knowledge, never achieved up to now in liquid solutions, with the exception of flash photolysis techniques.^[^ ^5^, ^6^ ^]^ However, in this case nitrate radicals are generated as a consequence of the impinging high energy radiation, while it is challenging to detect them when obtained from other processes of interest. In fact, nitrate radicals exhibit relatively short lifetimes and are often formed at low concentrations in liquid solutions. In particular, in water containing systems other radicals such as the hydroxyl ones can be simultaneously generated and could mask their presence, due to the comparable redox potential and the similar behavior in electron transfer and hydrogen abstraction reactions.^[^ ^7^ ^]^

Due to its paramagnetic nature, NO_3_^•^ should be detected via Electron Paramagnetic Resonance (EPR) technique. However, detection of this radical through EPR is well documented only in solid state, where it can be generated by high‐energy irradiation,^[^ ^8^, ^9^ ^]^ or in gas phase after reaction between oxygen atoms and NO_2_^•^,^[^ ^10^ ^]^ whereas, to the best of our knowledge, no EPR evidence has been ever reported up to now in liquid phase. Spin trapping methods, usually employed to form longer‐lived adducts that can be more easily detected by EPR, did not allow a clear attribution of the results to nitrate radicals. For instance, Nash et al.^[^ ^11^ ^]^ used DMPO (5,5‐dimethyl‐1‐pyrroline N‐oxide) as the trapping agent for nitrate radicals in aqueous solutions and observed the formation of DMPO‐OH as the hydrolysis product of the (only theoretically hypothesized) DMPO‐ONO_2_ adduct. Notably, DMPO‐OH can be formed by reaction of DMPO with other reactive oxygen species,^[^ ^12^ ^]^ which react faster than nitrate radicals with DMPO.^[^ ^13^ ^]^

For these reasons, the reaction of nitrate radicals with DMPO remains shrouded in ambiguity, strongly limiting the use of the powerful spin trapping techniques for the direct detection of this species.

Formation of nitrate radicals has been recently proposed to occur through oxidation of nitrate ions by the holes generated upon excitation of TiO_2_.^[^ ^3^ ^]^ However, the key role of nitrate radicals in these systems could not be supported by direct evidences due to the aqueous environment, but only postulated on the basis of indirect results. For instance, in irradiated TiO_2_ suspensions containing specific reactants and nitrate ions, by showing that the reaction products detected were obtainable only in the presence of nitrate radicals. One example is the oxidation of bromide ions into elemental bromine, which can be readily triggered by nitrate radicals but not by the reactive oxygen species (OH radicals, singlet oxygen and superoxide radical anions) traditionally reported in photocatalytic suspensions.^[^ ^14^, ^15^ ^]^ The same applies for the oxidative cleavage of the olefinic C═C bond producing the corresponding carbonyl compounds, which can be performed in non‐aqueous photocatalytic suspensions only hypothesizing the presence of nitrate radicals.^[^ ^16^, ^17^, ^18^, ^19^ ^]^ In the latter case, reaction takes place in acetonitrile as the solvent and in the presence of silver and nitrate ions. Silver ions efficiently scavenge photogenerated electrons, thus suppressing the formation of superoxide radical anions. Moreover, in the absence of water no hydroxyl radicals can be generated through the photogenerated holes, which mainly oxidize nitrate ions to nitrate radicals.

This photocatalytic system is therefore particularly useful to study the reactivity of nitrate radicals with DMPO. In fact, the interference of water and of reactive oxygen species, which would drive the reactivity of DMPO towards the more commonly observed DMPO‐OH, can be safely neglected.

We hereby report for the first time that using DMPO under these conditions allows detecting unambiguously nitrate radicals in non‐aqueous liquid suspensions. In fact, the detection of the stable radical species DMPOX, the oxidized form of the DMPO molecule, can be considered a fingerprint of the presence of nitrate radicals.

This finding not only represents a novel analytical method for the detection of nitrate radicals in liquid phase, but proves unambiguously their existence in photocatalytic systems, and supports the application of nitrate radicals in the field of green organic synthesis of industrially relevant compounds.

Following these considerations, DMPO was added to acetonitrile suspensions of TiO_2_, in the presence of silver nitrate (experimental details in Section S1). The reaction, taking place under UV‐A irradiation (Equations (1), (2), (3)), was monitored through EPR spectroscopy either under air or nitrogen atmosphere. Results are shown in Figure 1.

**Figure 1:**
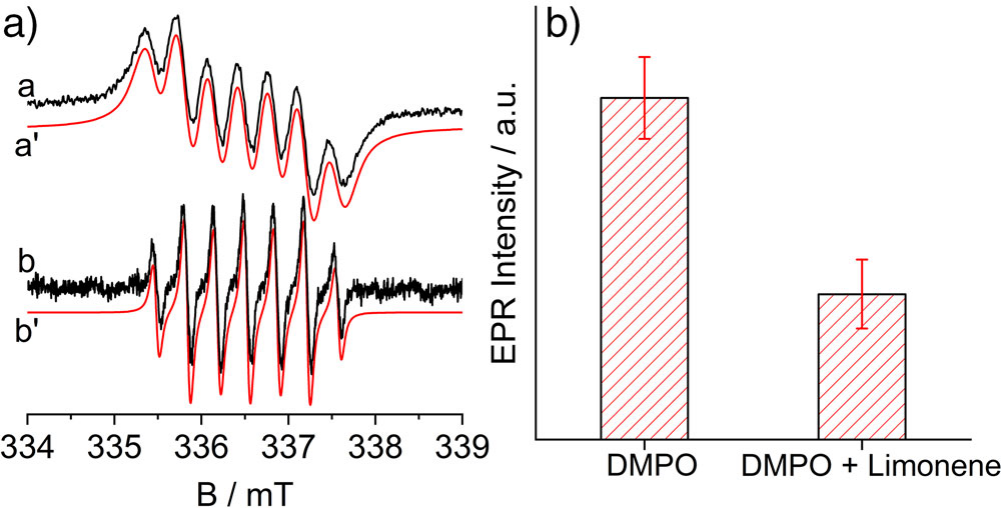
Panel a: EPR spectra of the DMPOX species produced via UV‐A light irradiation of DMPO in acetonitrile in the presence of TiO_2_ and AgNO_3_ in air (curve a) or under inert atmosphere (curve b), and the corresponding computer simulation (curve a’ and b’). Panel b: Signal intensity of the DMPOX in absence or in presence of limonene.

Upon UV‐A irradiation, the color of the photocatalytic suspension immediately turns from white to brown due to Ag^+^ reduction to metallic Ag, induced by the photogenerated electrons (see diffuse reflectance spectroscopy, transmission electron microscopy and X‐ray diffraction results in Section S2). Simultaneously, the EPR signal reported in Figure 1 (spectrum a) shows up. The signal is characterized by a very broad line shape and the spin Hamiltonian parameters extrapolated from the computer simulation (red lines in Figure 1) show a hyperfine coupling due to the nitrogen atom (nuclear spin I = 1) of about 0.69 mT and a further splitting of about 0.35 mT, due to two equivalent protons (I = ½) (Section S3, Figure S3). Such a hyperfine pattern is typical of the DMPOX radical species, the oxidized form of the DMPO trap molecule (Section S3, Figure S3). In the case of the DMPOX radical in fact, the reported hyperfine coupling for the unpaired electron with the nitrogen nucleus, is lower than that usually reported for the DMPO adducts (∼1.4 mT).^[^ ^20^, ^21^ ^]^

In order to exclude any possible oxidative contribution due to reactive oxygen species photocatalytically generated, the same test has been performed under inert atmosphere obtained bubbling N_2_ gas throughout the suspension during the whole experiment. Again, the DMPOX radical signal has been observed (Figure 1, spectrum b) but characterized by a sharper line shape. Such a difference is due to the absence of O_2_, which is known to cause line broadening as a consequence of its paramagnetic character (exchange narrowing effect).^[^ ^22^ ^]^ Therefore, the formation of DMPOX in deaerated conditions is strong evidence of the direct role of the NO_3_^•^. Notably, the same results are obtained in other common organic solvents (Section S4).

The simultaneous presence of an olefinic compound such as limonene in this system lowers the intensity of the observed signal (Figure 1b) indicating that limonene efficiently competes with DMPO for the generated nitrate radicals. This finding corroborates the key role played by nitrate radicals in the reported oxidative cleavage of olefins.^[^ ^16^, ^17^, ^18^, ^19^ ^]^ Accordingly, higher concentration of limonene completely suppresses the formation of DMPOX (Figure 2a). However, if O_2_ concentration is further increased by bubbling it through the suspension under the same conditions, the DMPOX signal shows up again (Figure 2b). These results can be rationalized by considering that at higher oxygen concentration much more photogenerated electrons are scavenged, as pointed out by the appearance of the DMPO‐O_2_^−^ adduct in the initial steps of the experiment of Figure 1b. The enhanced electron scavenging leads to a higher amount of NO_3_^•^ formed, thus favoring again the reaction with DMPO. Moreover, they further confirm the key role of silver ions as the electron scavengers for the observed reactivity.

**Figure 2:**
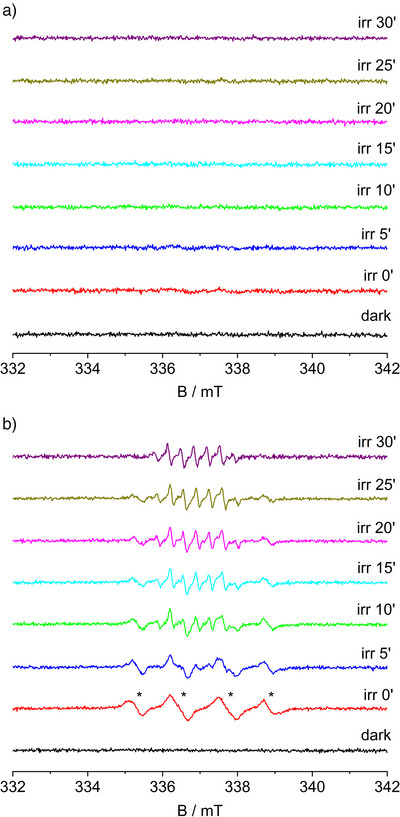
Spin trapping tests performed in concentrated suspensions in acetonitrile at different irradiation times, just after turning on the light source and under irradiation for 30 min. Reaction conditions: 10 mg of TiO_2_ P25, 34 mg of AgNO_3_ and 20 µL (123 µmol) of limonene were mixed in 0.5 ml of acetonitrile containing 22 µmol of DMPO. Panel a: reaction carried out in air. Panel b: reaction carried out bubbling O_2_. Asterisks indicate the spectroscopic features of the DMPO‐O_2_^−^ adduct.

Formation of DMPOX can be observed only when both Ag^+^ and the NO_3_^−^ ions are simultaneously present in the TiO_2_ suspension. In order to highlight the crucial role of their simultaneous presence by means of the spin trapping method, tests have been performed under inert atmosphere by separating the contributions of each of the two ions (Figure 3). The simple irradiation of a KNO_3_ or LiNO_3_ solution in presence of TiO_2_ and the DMPO trap does not lead to the detection of any kind of radicals (Figure 3a). In the absence of nitrate ions, AgClO_4_ was employed as the source of Ag^+^ ions. The solution was irradiated for 20 min; the color of the suspension turned immediately brown because of the formation of metallic silver, but no EPR signals were detected (Figure 3b). After turning off the light source, the suspension was kept in dark for 20 min and an EPR spectrum was recorded to exclude any trivial redox reaction involving perchlorate ions or silver nanoparticles under dark conditions (Figure 3c). Then NO_3_^−^ ions were added to the suspension in dark and again no EPR signals were detected (Figure 3d). Thereafter, the suspension was irradiated by UV‐A light and in this case the DMPOX signal showed up again (Figure 3e). These experimental results clearly indicate the photocatalytic origin of nitrate radicals and show that the spin trapping method is a powerful technique for their detection.

**Figure 3:**
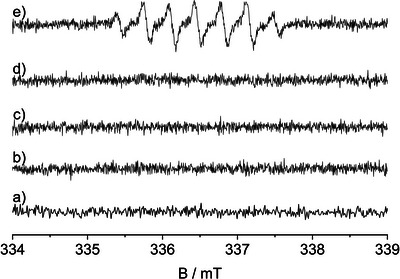
Spin trapping test performed under inert atmosphere (N_2_), under UV‐A irradiation, and in the presence of different inorganic salts. a) LiNO_3_; b) AgClO_4_; c) AgClO_4_ after turning off the UV‐A light; d) adding of LiNO_3_ in dark; e) turning on the irradiation in presence of both AgClO_4_ and LiNO_3_.

An important evidence of the reactivity of NO_3_^•^ with unsaturated organic substrates is the release of NO_2_^•^,^[^ ^16^, ^17^, ^18^, ^19^ ^]^ and it is reported that the DMPOX can be formed by reaction of DMPO with this latter species.^[^ ^23^ ^]^ In order to clarify the role of NO_2_^•^ in our system, a representative run was performed under a continuous oxygen flow and by bubbling the gas phase exiting the irradiated suspension through a Griess solution, which becomes pink‐red in the presence of NO_2_^•^. The absorbance of the dye formed by the azo‐coupling Griess reaction, was measured by means of UV‐Vis spectroscopy (*λ*_max_ = 549 nm) and correlated to the amount of NO_2_^•^ produced. Simultaneously, EPR spectra were recorded to monitor the formation of DMPOX. Results are shown in Figure 4, along with the results of a blank test (red line) performed in the absence of DMPO. Experimental details are reported in Sections S5 and S6.

**Figure 4:**
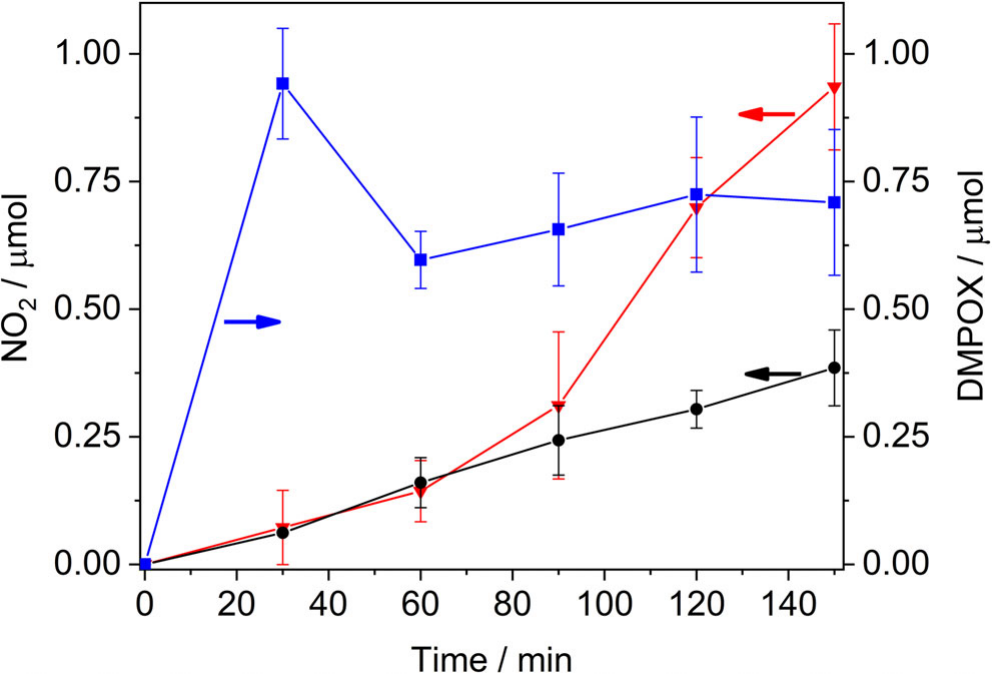
NO_2_^•^ evolution versus time in presence of DMPO (black curve) and without DMPO (red curve). In the former case DMPOX generation was also evaluated (blue curve).

Notably, NO_2_^•^ was detected both in the presence (Figure 4 black line) and in the absence of DMPO (Figure 4 red line). To the best of our knowledge, photocatalytic nitrate reduction to NO_2_^•^ has never been reported in literature under aerated conditions. However, Zou et al.^[^ ^24^ ^]^ report that in presence of AgNO_3_ acetonitrile can react with NO_3_^•^ photolytically generated in homogeneous phase forming CH_3_ONO_2_ and CN^•^ (Equation 4). The CN^•^ in turn forms AgCN (Equation 5) and the CH_3_ONO_2_ easily decomposes under UV irradiation releasing NO_2_^•^.^[^ ^25^, ^26^, ^27^ ^]^

The whole photocatalytic reaction can be now summarized by the following processes.

Charge carriers are photogenerated (Equation 1) and scavenged by the Ag^+^ and NO_3_^−^ ions (Equations 2 and 3).

(1) $$\mathrm{Ti}O_{2}+h\nu \to \mathrm{Ti}O_{2}\left(h^{+},e^{-}\right)$$

(2) $$Ag^{+}+e^{-}\to Ag^{0}$$

(3) $$N{O_{3}}^{-}+h^{+}\to N{O_{3}}^{•}$$

In the absence of DMPO, the reaction with acetonitrile occurs (Equations 4 and 5). Accordingly, AgCN was detected by XRD analysis of the recovered photocatalyst only in the absence of DMPO (Section S2, Figure S2).

(4) $$N{O_{3}}^{•}+CH_{3}\mathrm{CN}\to CH_{3}\mathrm{ON}O_{2}+CN^{•}$$

(5) $$Ag^{0}+CN^{•}\to \mathrm{AgCN}$$

In the presence of DMPO, DMPOX is quantitatively generated in the first part of the experiment (Figure 4, blue line) when only a tiny amount of NO_2_^•^ is formed (Figure 4, black line). This indicates that, even though the reaction of DMPO with NO_2_^•^ is reported in aqueous media,^[^ ^23^, ^28^ ^]^ in the present case such a process can be neglected. Therefore, it can be assumed that the observed NO_2_^•^ derives from the reaction of DMPO with nitrate radicals.

In this case, although the detailed reaction mechanism is under investigation, the evidence of NO_2_^•^ generation allows to draw the following considerations about the process forming the DMPOX. Due to the spin conservation rule, a direct generation of a second radical species beside the DMPOX can be excluded. For this reason, the reaction process has to involve either the formation of a diamagnetic product as HNO_2_ (Equation 6), or a more complex pathway involving for instance two nitrate radicals (Equation 7), similarly to what reported for the C═C cleavage induced by nitrate radicals.^[^ ^16^, ^17^, ^18^, ^19^ ^]^ The release of NO_2_^•^ however, suggests that the reaction pathway is better described by Equation 7.

(6) $$\mathrm{DMPO}+N{O_{3}}^{•}\to \mathrm{DMPOX}+\mathrm{HN}O_{2}$$

(7) $$\mathrm{DMPO}+2N{O_{3}}^{•}\to \mathrm{DMPOX}+N{O_{2}}^{•}+\mathrm{HN}O_{3}$$

By concluding, the present experimental results (i) represent the first spectroscopic evidence of nitrate radicals in solution by means of EPR spectroscopy, (ii) clarify the reactivity of nitrate radicals with a commonly used spin trap agent such as DMPO, and (iii) indicate that spin trapping method with DMPO is a suitable way to directly and semi‐quantitatively highlight the generation of nitrate radicals in complex non‐aqueous reacting media.

## Supporting Information

The authors have cited additional references within the Supporting Information.^[20–22]^

## Conflict of Interests

The authors declare no conflict of interest.

## Supporting information



Supporting Information

## Data Availability

The data that support the findings of this study are available from the corresponding author upon reasonable request.
