# Supplementary material for: Reaction of Nitrate Radicals and DMPO in Non‐Aqueous Media: A Novel Detection Tool to Highlight the Nitrate Radical Role in Photocatalytic Processes
Source: Angew Chem Int Ed Engl. 2025 Apr 2;64(23):e202505313. doi: 10.1002/anie.202505313 (PMC12124427; doi:10.1002/anie.202505313)
Supplement: Supplementary file 1 — Supporting Information [file ANIE-64-e202505313-s001.docx]

SUPPORTING INFORMATION

Reaction of Nitrate Radicals and DMPO in Non-aqueous Media: A Novel Detection Tool to Highlight the Nitrate Radical Role in Photocatalytic Processes.

Alessia Zollo,^[a]^ Alessandro Gottuso,^[b]^ Leonardo Palmisano,^[c]^ Elio Giamello,^[a]^ Francesco Parrino,*^[b]^ and Stefano Livraghi*^[a]^

[a]	Dr. A. Zollo, Prof. E. Giamello, Prof. S. Livraghi
Department of Chemistry and NIS Centre

University of Torino

Via Pietro Giuria 7, 10125 Torino, Italy
 E-mail: stefano.livraghi@unitn.it

[b]	Dr. A. Gottuso, Prof. F. Parrino
Department of Industrial Engineering
University of Trento
Via Sommarive 9, 38123 Trento, Italy

E-mail: francesco.parrino@unitn.it

[c]	Prof. L. Palmisano

Department of Engineering

University of Palermo

Viale delle Scienze 9, Ed. 6, 90128 Palermo, Italy

**S1. Materials and methods**

*S1.1 Materials*

Acetonitrile (CH_3_CN, d = 0.780 g/mL, anhydrous, 99.8%), silver nitrate (AgNO_3_, ≥ 99.0%), lithium nitrate (LiNO_3_, 99.99%), silver perchlorate (AgClO_4_, anhydrous, 97%), 5,5-Dimethyl-1-pyrroline N-oxide (DMPO, ≥ 98.0%), and (*R*)-limonene (C_10_H_16_, d = 0.844 g/mL, 97%) were purchased from Sigma Aldrich. TiO_2_ P25 (specific surface area ca. 50 m^2^/g; anatase (73-85 %), rutile (14-17 %), amorphous (0-13 %))^[20]^ was purchased from Evonik. All of the materials were used as received without further purification.

*S1.2 Experimental details of the reaction system*

Spin trapping experiments have been performed by adding amounts of AgNO_3_ and limonene used in a previous work.^[8]^ Notably, 34 mg of AgNO_3_ were dissolved in 20 ml acetonitrile in the presence of 10 mg of TiO_2_ and 250 µl of a 0.088 M solution of DMPO. Selected runs have been performed in the presence of limonene (1.23 mM) and/or bubbling nitrogen or oxygen through the suspension. The suspension was kept in the dark for 30 minutes before UV-A irradiation performed with a LED centred at 360 nm (irradiance value 125 W/m^2^).

*S1.3 Characterization methods*

Spin trapping experiments have been followed on an ADANI SPINSCAN X spectrometer operating at an X-band (9.5 GHz). The suspension was collected with a capillary and measured at room temperature. In all cases, the EPR modulation amplitude was 0.1 mT and the microwave power 10 mW.

The photocatalytic material after selected runs (30 minutes irradiation) was recovered by centrifugation, dried at 70°C and characterized as below detailed.

X-ray diffraction (XRD) patterns of the powders recovered after the reaction were obtained from a PANalytical PW3040/60 X'Pert PRO MPD diffractometer using a copper Kα radiation source. The diffraction patterns were obtained in the 2θ range between 20° and 50° using an 8 mm capillary method and the X'Pert High-Score software was used for data handling.

High-resolution Transmission Electron Microscopy (HRTEM) images were obtained on a JEOL 3010-UHR instrument (acceleration potential: 300 kV) equipped with an EDAX probe. Samples for TEM investigation were supported onto a holed carbon coated copper grid by dry deposition.

UV-Vis diffuse reflectance spectra (DRS) were recorded by using a Varian Cary 5000 spectrometer. A Polytetrafluoroethylene (PTFE) sample was used as the reference. The spectrum was recorded in the 200–800 nm range at a scan rate of 240 nm/min with a step size of 1 nm. The measured reflectance was converted with the Kubelka-Munk function.

**S2. Characterization results of the catalyst recovered after reaction**

HR-TEM and EDX analyses of the recovered photocatalyst after a representative run are shown in Figure S1 a-c, while the corresponding diffuse reflectance spectrum is reported in Figure S1 d. Results are similar regardless of the presence of limonene and DMPO. Silver nanoparticles of few nanometers, along with some bigger aggregates are visible at the surface of TiO_2_. The diffuse reflectance UV-Vis spectrum of the same sample besides the band to band transition in the UV region due to TiO_2_, shows the typical localized surface plasmon resonance (LSPR) induced absorption band of silver nanoparticles with a maximum centred at ca. 480 nm. Accordingly, the suspension became brownish upon UV-A irradiation.

**Figure S1.** a: HR-TEM image of the TiO_2_ photocatalyst recovered after 180 min of UV-A irradiation in the presence of AgNO_3_ 10 mM and (*R*)-limonene 2 mM in acetonitrile. Silver nanoparticles (Ag NPs) are highlighted in yellow; b: EDX map of titanium atoms (light blue points); c: EDX map of silver atoms (green points); d: UV-Vis diffuse reflectance spectrum (DRS) of the same sample.

Figure S2 shows the XRD patterns for the powders recovered after the photocatalytic reaction without DMPO (pattern a) and in presence of DMPO (pattern b). In both cases, as expected, residual AgNO_3_ and metallic silver are observed. Concerning the residual AgNO_3_ it is worth to mention that in the two experiments the salt has crystallized in two different crystallographic structures: when DMPO is not present it crystallizes in the orthorhombic lattice, whereas in presence of DMPO in the rhombohedral form. Silver nitrate in fact shows complex and unusual polymorphic behaviour, the most common structure at normal temperature and pressure is the orthorhombic, but other phases including the rhombohedral are also reported.^[21]^

Whatever the crystallization form of AgNO_3_, the most important finding in the two cases is the presence of AgCN only in the absence of DMPO. The presence of AgCN in fact clearly indicates that in this case the reaction between CH_3_CN and NO_3_^•^ radicals occurs. When DMPO is present, due to the competition with the NO_3_^•^ radicals, AgCN is not formed.

**Figure S2.** XRD patterns (top) of the powders recovered after the photocatalytic reaction performed in presence (a, red) and in absence (b, black) of DMPO. In the lower part of the panel the reference XRD patterns are shown: Anatase (00-021-1272), Rutile (01-076-0318), AgNO_3_ in rhombohedral form (01-070-0198), AgNO_3_ in orthorhombic form (00-043-0649), metallic silver (00-004-0783) and, highlighted in red, AgCN (00-023-1404).

**S3. EPR spectrum of DMPOX**

Figure S3 reports the computer simulation of the DMPOX spectrum together with the stick diagram to better clarify the hyperfine splitting pattern due to the couplings of the unpaired electron with the N and H nuclei. Computer simulation was obtained employing the SIM32 program.^[22]^

**Figure S3.** Experimental (black) and simulated (red) EPR spectrum of DMPOX. The experimental signal was collected under inert atmosphere. The stick diagram shows the hyperfine couplings of the unpaired electron with the N nucleus (0.69 mT) and with the two hydrogens nuclei in γ position (both 0.35 mT). In the lower part of the Figure, chemical structures of DMPO and DMPOX.

**S4. Nitrate radical detection in other solvents**

**Figure S4.** Experimental (black) and simulated (red) EPR spectra of DMPOX in DMSO (panel A) and THF (panel B). The experimental signal was collected under inert atmosphere.

Figure S4 shows the DMPOX signal acquired performing the same experiment in two other solvents, dimethylsulfoxide (DMSO, panel A) and tetrahydrofuran (THF, panel B). The reported computer simulation (in red) allows the determination of the hyperfine couplings with the nitrogen and the hydrogen nuclei in γ position, which as expected, are similar to the ones previously reported for DMPOX in acetonitrile.

**S5. Quantification of DMPOX**

DMPOX produced during the reaction in non-concentrated conditions (10 mg of TiO_2_, 34 mg AgNO_3_ in 20 ml acetonitrile containing this time 1 ml of a 0.088 M DMPO solution) was quantified building a calibration curve (Figure S5) with the standard DPPH (2,2-diphenyl-1-picrylhydrazyl), evaluating the area of the EPR signal for each concentration. The EPR measurements of DMPOX were carried out collecting the suspension from the reactor with a capillary every 30 minutes for a total time of 150 minutes (Figure 3 main text, blue curve).

**Figure S5.** Calibration curve obtained from the EPR signals areas of DPPH (R^2^ = 0.999).

**S6. Griess tests for NO_2_ detection**

The amount of NO_2_ generated during the reaction carried out in non-concentrated conditions was monitored by bubbling nitrogen through the suspension and by flowing the gas phase exiting the reactor in 50 ml of a Griess solution, as detailed below. This test has been performed both in the presence and in the absence of DMPO. When present, 1 ml of DMPO 0.088 M was added to the flask.

The quantity of developed NO_2_ was assessed by measuring the absorbance of the Griess reagent, which turns pink-red in the presence of NO_2_ or NO_2_^-^. Said reagent consists of an acidic solution of sulfanilic acid and N-(1-naphtyl)ethylendiamine as coupling agents. The reaction is described according the Eqs. S1 and S2.

 (S1)

(S2)

In the initial step, the nitrite ion reacts with the amine group of sulfanilic acid to form a diazonium salt (Eq. S1). This salt then undergoes an azo-coupling reaction with N-(1-naphthyl)ethylenediamine (Eq. S2), resulting in the formation of a pink-red azo dye. The concentration of this dye can be determined through UV-Vis spectroscopy, with its absorbance directly linked to the quantity of nitrite ions present.

To prepare the Griess reagent, 0.25 g of sulfanilic acid (99%, Sigma-Aldrich), 7 mL of glacial acetic acid (≥ 99%, Sigma-Aldrich), and 1 mL of a 1 g/L aqueous solution of N-(1-naphthyl)ethylenediamine dihydrochloride (98%, Sigma-Aldrich) were combined in flask and brought to 50 ml with deionized water. The mixture was sonicated for 10 minutes and used as a trap for the gas stream exiting the photocatalytic reactor, causing the solution to turn pink-red. The absorbance of the coloured solution was measured at 549 nm using a UV-Vis spectrophotometer (Ossila spectrometer G2001A1, Leiden, The Netherlands) and correlated to the NO₂ content in the gas stream using a calibration curve (Figure S6), considering that the NO_2_ : NO_2_^-^ ratio is 2 : 1 according to the reaction:

2NO_2_ + H_2_O 🡪 HNO_3_ + HNO_2_	(S3)

The calibration curve was built adding various amounts of a 0.5 mM NaNO₂ solution to 5 ml of the Griess solution. The pink-red colour that developed after 15 minutes was analyzed via UV-Vis spectroscopy.

**Figure S6.** Calibration curve obtained by considering the absorbance at λ_max_ = 549 nm (R^2^ = 0.997)

**Additional References**

[20] B. Othani, O.O. Prieto-Mahaney, D. Li, R. Abe*, Journal of Photochemistry Photobiol*ogy *A* **2010**, *216*, 179-182.

[21] D.M. Adams, S.K. Sharm, *Journal of the Chemical Society, Faraday Transactions 2: Molecular and Chemical Physics* **1976**, *72*, 848-854.

[22] A. Adamski, T. Spalek, Z. Sojka, *Research on Chemical Intermediates* **2003**, *29 (7)*, 793–804.
